# Generalized Fractional Derivative Anisotropic Viscoelastic Characterization

**DOI:** 10.3390/ma5010169

**Published:** 2012-01-18

**Authors:** Harry H. Hilton

**Affiliations:** Aerospace Engineering Department, College of Engineering and Private Sector Program Division, National Center for Supercomputing Applications (NCSA), University of Illinois at Urbana-Champaign (UIUC), 104 S. Wright Street, MC-236, Urbana, IL 61801–2935, USA; E-Mail: h-hilton@illinois.edu; Tel.: +1-217-840-1116; Fax: +1-217-244-0720

**Keywords:** anisotropic non-homogeneous viscoelasticity, error analysis, fractional derivatives, functionally graded materials, generalized Kelvin models, master relaxation curves, material characterization, nonlinear viscoelastic properties, shift functions

## Abstract

Isotropic linear and nonlinear fractional derivative constitutive relations are formulated and examined in terms of many parameter generalized Kelvin models and are analytically extended to cover general anisotropic homogeneous or non-homogeneous as well as functionally graded viscoelastic material behavior. Equivalent integral constitutive relations, which are computationally more powerful, are derived from fractional differential ones and the associated anisotropic temperature-moisture-degree-of-cure shift functions and reduced times are established. Approximate Fourier transform inversions for fractional derivative relations are formulated and their accuracy is evaluated. The efficacy of integer and fractional derivative constitutive relations is compared and the preferential use of either characterization in analyzing isotropic and anisotropic real materials must be examined on a case-by-case basis. Approximate protocols for curve fitting analytical fractional derivative results to experimental data are formulated and evaluated.

## 1. Introduction

A vast body of literature indicates that the predominant choice in viscoelastic research, analysis and design is for constitutive relations consisting of integral relations, which are the Green’s functions associated with integral order differential equations [1,2,3,4,5]. However, an alternative representation for isotropic viscoelasticity in terms of fractional derivative calculus has been developed in [6,7,8,9,10,11,12,13,14] and generalized [15]. This concept, which is reflected in a substantial number of publications, is based on the premise that the derivatives in viscoelastic constitutive relations need not necessarily be of integer order, but may be of some fractional order with similar translations to integral representations. The mathematical foundation of fractional calculus and of the attending Laplace and Fourier transforms is well developed and may be found in [16,17,18,19,20,21,22,23], to mention only a few. In [24] a comprehensive historical review of linear isotropic viscoelastic characterizations based on fractional derivatives has been presented.

Volterra [25] was the first to extend Boltzmann’s isotropic superposition principle [26] to anisotropic viscoelasticity. Subsequently, in [27,28] anisotropic viscoelastic stress-strain relations were formulated. The mathematical and physical foundations of anisotropic viscoelasticity firmly rest on the well established isotropic viscoelastic concepts as shown in [29,30]. The elastic-viscoelastic integral transform correspondence principle (analogy) was extended to anisotropic viscoelasticity and latter’s proper mechanical modeling was formulated in [31]. In [31] it was shown that the powerful tool of separation of variables available under proper conditions in isotropic viscoelasticity is inadmissible in anisotropic viscoelasticity, leaving only Laplace (LT) and Fourier (FT) transforms, finite element methods (FEM) and a limited number of analytical solutions such as asymptotic approaches capable of producing analytical anisotropic solutions. Presently, the main applications of anisotropic viscoelasticity are in the areas of fiber/high polymer composites, fiber reinforced concrete, filament wound vessels, textiles and tires.

In the present paper the concept of single fractional derivative isotropic viscoelastic constitutive relations is formalized for anisotropic viscoelasticity in terms of many parameter generalized Kelvin models. Both differential and integral stress-strain equations and expressions for the associated complex moduli, compliances and creep functions are derived and their applicability and usefulness are evaluated. Viscoelastic integral stress-strain laws are always preferable to differential ones, because they are relatively easier to handle and computationally yield more accurate results.

## 2. Analysis

### 2.1. Material Property Dependence on Temperature

**Figure 1:**
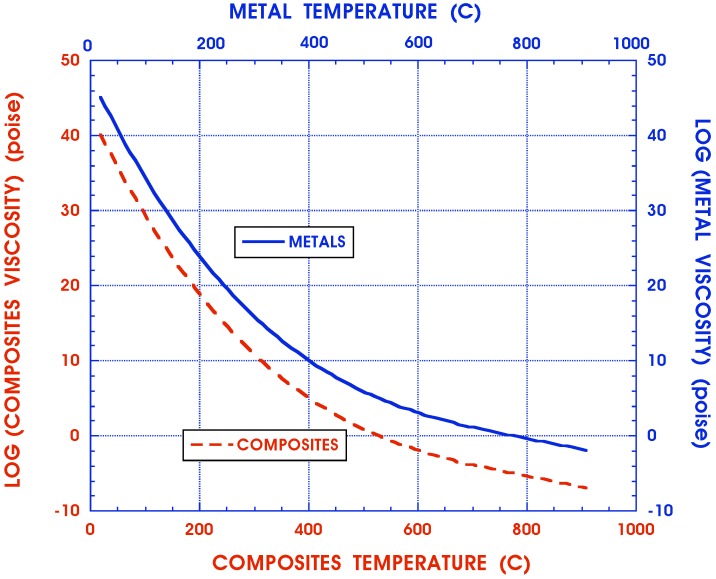
Coefficients of viscosity for metals and composites.

Viscoelastic media, be they metals at elevated temperatures [32,33] or high polymers [34], are well known to exhibit material properties that are extremely temperature sensitive. In general, their coefficients of viscosity vary one order of magnitude for roughly every 20 °C (Figure 1), which is described by the Arrhenius relation

(1) $$\eta \;=\;\eta_{0}\;\exp\left(-\frac{Q_{a}}{R\;\Theta }\right)$$

where $Q_{a}$ is the activation energy, *R* the universal gas constant and Θ the material temperature in degrees Kelvin.

Consider a Cartesian system $x=\{x_{1},x_{2},x_{3}\}$. The linear viscoelastic constitutive relations may be written in the form:

(2) $$\left\{\sigma (x,t)\right\}\;=\;\int_{-\infty }^{t}\left[E(x,t,t^{\prime })\right]\;\left\{\frac{\partial \epsilon (x,t^{\prime })}{\partial t^{\prime }}\right\}\;dt^{\prime }\;-\;\int_{-\infty }^{t}\left[E^{T}\left(x,t,t^{\prime }\right)\right]\;\left\{\frac{\partial [\alpha T\left(x,t^{\prime }\right)]}{\partial t^{\prime }}\right\}\;dt^{\prime }$$

These relations may be then classified into categories as seen in Table 1. Consequently, the first three cases are relatively “simple” integral relations, whereas the third one, as will be seen subsequently, presents considerable difficulties. These issues will be analyzed in detail in the next Sections.

**Table 1:** Temperature influence on constitutive relations.

Temperature	Modulus	Type of Constitutive Relations, Equation (2)	Type of Material
$T_{0}$	$E(t-t,\;\;{}^{\prime }\;T_{0})$	convolution	homogeneous
$T_{0}$	$E(x,t-t,\;\;{}^{\prime }\;T_{0})$	convolution	non-homogeneous
T(x)	$E[x,t-t,\;\;{}^{\prime }\;T\left(x\right)]$	convolution	non-homogeneous
T(x,t)	$E[x,t,t,\;\;{}^{\prime }\;T\left(x,t^{\prime }\right)]$	non-convolution	non-homogeneous

### 2.2. General Concepts—Isotropic Materials

In order to systematically formulate anisotropic viscoelastic fractional derivative constitutive relations, it is necessary to first make a few general comments about isotropic relations with constant $\left(T_{0}\right)$ and spatially varying $\left(T(x\right))$ temperatures (see Table 1). In [15] generalized isotropic fractional derivative (FD) Maxwell models (GFDMM) are examined, while in [1,2,3] integer derivative (ID) differential and integral equations (DE and IE) for isotropic viscoelasticity in terms of generalized Maxwell and Kelvin models (GMM and GKM). A parallel development for integer order derivative DE and IE anisotropic viscoelastic responses has been formulated in [35]. In general, even though such modeling representations are interrelated and, therefore, interchangeable, GMM are better suited for numerical analyses, while GKM are more useful for achieving mathematical and physical understanding of material behavior. The use of GKM or GMM and GFDKM or GFDMM guarantees that any additive combination automatically satisfies thermodynamic principles.

**Figure 2:**
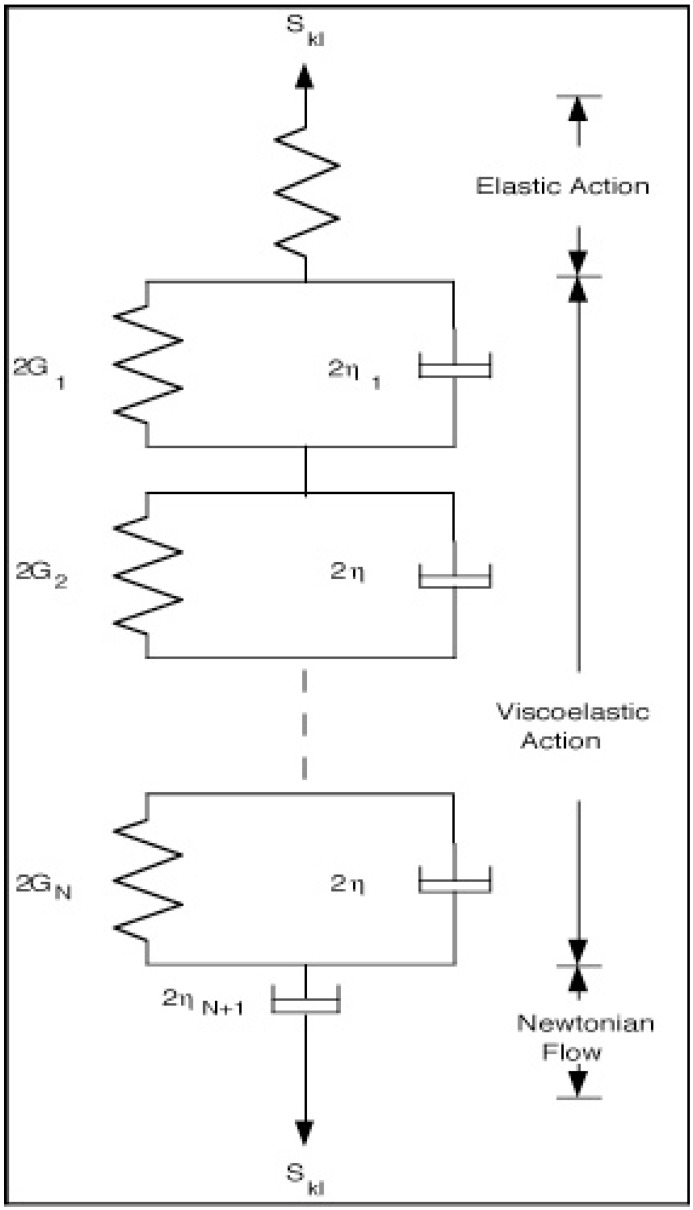
Generalized Kelvin Model.

Consider a GKM or GFDKM as shown in Figure 2. The single spring represents instantaneous elastic responses associated with “solids” and the single dashpot represents long time behavior tending to large or infinite displacements for long time periods. For isotropic cases, it is most useful to write separate constitutive relations for changes in shape and in volume, since real materials behave distinctly during these changes. Using Einstein’s Cartesian tensor notation and summation convention, all relations to follow are formulated in terms of non-dimensionalized material parameters and state variables. The mean stresses *σ* and strains *ϵ* are defined as

(3) $$\sigma \;=\;\sigma_{ii}/3\;\;\;\;\;\;\mathrm{and}\;\;\;\;\;\;\epsilon \;=\;\epsilon_{ii}/3$$

with stress and strain deviators

(4) $$S_{kl}\;=\;\sigma_{kl}\;-\;\delta_{kl}\;\sigma \;\;\;\;\;\;\mathrm{and}\;\;\;\;\;\;E_{kl}\;=\;\epsilon_{kl}\;-\;\delta_{kl}\;\epsilon$$

The various elements of a GFDKM (Figure 2) have the following constitutive relations

(5) $$\begin{array}{cc} \text{elastic}\;\;\text{spring}\;⟹\;S_{kl}\; & =\;2\;G_{0}\;E_{kl}^{\left(o\right)} \end{array}$$

(6) $$\begin{array}{cc} \text{each}\;\;\text{FDKM}\;⟹\;S_{kl}\; & =\;\underset{n^{\mathrm{th}}\;\mathrm{dashpot}}{\underset{︸}{2\;\eta_{n}\;D^{\delta_{n}}E_{kl}^{\left(n\right)}}}\;+\;\underset{n^{\mathrm{th}}\;\mathrm{spring}}{\underset{︸}{2\;G_{n}E_{kl}^{\left(n\right)}}}\;\;\;\;\;\;\left(1\leq n\leq N\right) \end{array}$$

(7) $$\begin{array}{cc} \text{single}\;\;\text{dashpot}\;⟹\;S_{kl}\; & =\;2\;\eta_{N+1}\;D^{\delta_{N+1}}E_{kl}^{\left(N+1\right)} \end{array}$$

where $\delta_{n}$ are distinct nonintegers and the derivatives $D^{\delta_{n}}=d^{\delta_{n}}/dt^{\delta_{n}}$ are defined in the Fourier sense [36]. For $\delta_{n}=1$ with 1≤n≤N+1, these fractional derivative (FD) representations immediately reduce to the corresponding integer derivative (ID) ones. Alternatively, without changing any of the relations to follow, the FDs can also be represented in the Riemann–Liouville format [37] as

(8) $$D^{\delta_{n}}\left\{F\left(t\right)\right\}\;=\;\frac{d^{\delta_{n}}F\left(t\right)}{dt^{\delta_{n}}}\;=\;\frac{1}{\Gamma (1-\delta_{n})}\;\frac{d}{dt}\int_{0}^{t}\frac{F(t^{\prime })}{{\left(t-t^{\prime }\right)}^{\delta_{n}}}\;dt^{\prime }\;\;\;\;\;\;\left(0<\delta_{n}<1\right)$$

but with the limitations $\delta_{n}<1<n$ it is not possible to recover the corresponding integer derivatives $d^{n}/dt^{n}$. However, the original definitions in [38,39,40] carried no such restrictions, requiring only that $ℜ(\delta_{n})>0$. In the present paper, the original more permissive condition for $\delta_{n}$ will be enforced.

The total strain deviator for the GFDKM and also for the GKM is the sum of the individual contributions, or

(9) $$E_{kl}\;=\;\sum_{n=0}^{N+1}E_{kl}^{\left(n\right)}$$

while this sum may be formed by successive fractional differentiations of the individual parts [3], it is much more expeditious to apply FT to Equations (5)–(7), such that

(10) $$2{\bar{\bar{E}}}_{kl}\;=\;\left[\frac{1}{G_{0}}\;+\;\frac{1}{\eta_{N+1}{\left(ı\;\omega \right)}^{\delta_{N+1}}}\;+\;\sum_{n=1}^{N}\frac{1}{\eta_{n}}\;\frac{1}{{\left(ı\;\omega \right)}^{\delta_{n}}+1/\tau_{n}}\right]{\bar{\bar{S}}}_{kl}\;=\;\frac{{\bar{\bar{S}}}_{kl}}{{\bar{\bar{G}}}_{FD}}$$

where $ı=\sqrt{-1}$ and the double bars indicate FT. From Equation (10), the complex shear modulus then can be defined as

(11) $$\frac{1}{{\bar{\bar{G}}}_{FD}}\;=\;\frac{\prod_{n=1}^{N+1}\left[A_{n}\;{\left(ı\;\omega \right)}^{\gamma_{n}}\;+\;B_{n}\right]}{G_{0}\;{\left(ı\;\omega \right)}^{\delta_{N+1}}\prod_{n=1}^{N}\left[{\left(ı\;\omega \right)}^{\delta_{n}}\;+\;1/\tau_{n}\right]}\;=\;{\bar{\bar{J}}}_{FD}$$

with ${\bar{\bar{J}}}_{FD}$ as the complex shear compliance. The complex moduli and compliances, of course, are at the heart of the isotropic and anisotropic elastic-viscoelastic integral transform analogies [3,35].

Substitution of Equation (11) into (10) and formally carrying out the multiplications results in

(12) $$2G_{0}\sum_{n=0}^{N+1}b_{n}^{\prime }\;{\left(ı\;\omega \right)}^{\beta_{n}}\;{\bar{\bar{E}}}_{kl}\;=\;\sum_{n=0}^{N+1}a_{n}^{\prime }\;{\left(ı\;\omega \right)}^{\alpha_{n}}\;{\bar{\bar{S}}}_{kl}$$

and upon inverting the FT yields

(13) $$2G_{0}\;Q_{FD}\left\{E_{kl}\right\}\;=\;P_{FD}\left\{E_{kl}\right\}$$

where

(14) $$P_{FD}\;=\;\sum_{n=0}^{r}a_{n}^{\prime }\;D^{\alpha_{n}}\;\;\;\;\;\;\text{and}\;\;\;\;\;\;Q_{FD}\;=\;\sum_{n=0}^{s}b_{n}^{\prime }\;D^{\beta_{n}}$$

It should be noted that when $G_{0}<\infty$ then the single elastic spring is present in the system. (See [3] for other combinations and for the relations between *r*, *s* and *N*.) Some other observations are noteworthy, namely that for $G_{0}<\infty$ and r=s=N+1

(15) $$\alpha_{N}\;=\;\beta_{N}\;=\;\sum_{n=1}^{N+1}\delta_{n}$$

and with the single dashpot absent, $\delta_{N+1}=0$ or $\eta_{N+1}=\infty$ or both. For $\alpha_{n}=\beta_{n}=n$, the fractional derivative operators $P_{FD}$ and $Q_{FD}$ reduce to integer order derivative ones denoted by *P* and *Q*. On the other hand, the fractional derivative orders $\alpha_{n}$ are not necessarily equal to the corresponding $\beta_{n}$ for each *n*. One of the advantages of the fractional derivative models is that for the same number *N* of individual Kelvin or Maxwell bodies a third more material parameters, *i.e.*, the $\delta_{n}$, are available for fitting actual creep or relaxation data, thus in principle, reducing the needed value of *N* for the same goodness of fit when compared to ID characterizations.

**Figure 3:**
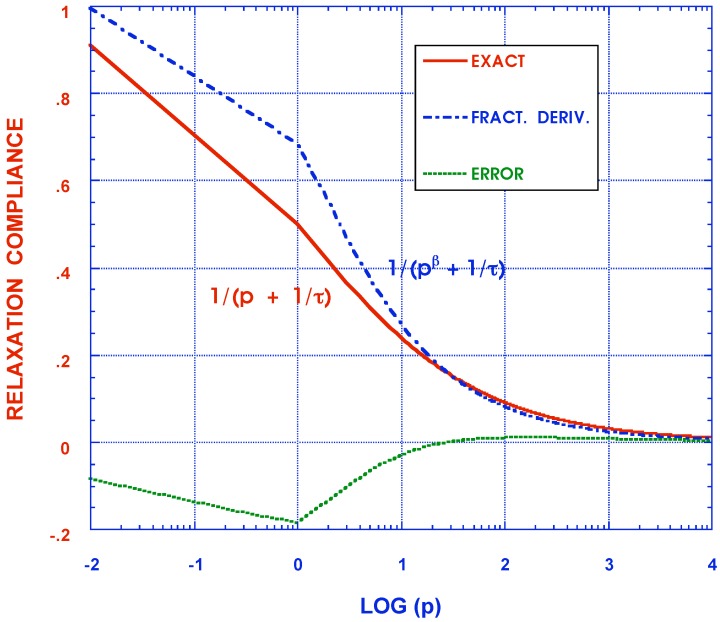
Comparison of exact and approximate relaxation moduli Laplace transforms of $1/(p^{\beta }+1/\tau )$ and $1/{\left(p+c\right)}^{\mu }$.

The FT of the creep and relaxation functions are given by

(16) $$ı\;\omega {\bar{\bar{\phi }}}_{FD}\;=\;\frac{1}{ı\;\omega {\bar{\bar{\psi }}}_{FD}}\;=\;{\bar{\bar{G}}}_{FD}\;=\;\frac{1}{{\bar{\bar{J}}}_{FD}}$$

which relates the FT of the creep function ${\bar{\bar{\psi }}}_{FD}$ directly to the expression in square bracket in Equation (10). The FT inversion of the first two terms poses no difficulty, but the individual terms in the summation have no tabulated transform. Consequently, their inversion can be accomplished only by formal integration, fast Fourier transforms (FFT) [41], asymptotic expansions or some approximation. In [15] one of these for a single β= .5 and large times are presented. In [11] the approximation applicable for long times, *i.e.*, small *ω*s,

(17) $$\frac{1}{1+{\left(ı\;\omega \right)}^{\beta }\;\tau }\;≃\;1-{\left(ı\;\omega \right)}^{\beta }\;\tau \;\;\;\;\;\;\mathrm{for}\;\;{\left(ı\;\omega \right)}^{\beta }\;\tau \ll 1\;\mathrm{and}\;t\gg 1$$

has been used. Alternatively, a different approximate inversion scheme is suggested here for each fraction in the sum of Equation (10) by noting the near similarity of the functions

(18) $$\frac{1}{{\left(ı\;\omega \right)}^{\delta_{n}}+1/\tau_{n}}\;≃\;\frac{1}{{\left(ı\;\omega +c_{n}\right)}^{\mu_{n}}}\;⟹\;F\left(t\right)\;=\;\frac{e^{-c_{n}t}\;t^{\mu_{n}-1}}{\Gamma (c_{n})}$$

as seen in Figure 3 with p=ıω for corresponding Laplace transforms. While the agreement is poorest in the range of small values of *p*, it must be remembered that this is the region of very long time responses where solutions may not be required and where asymptotic expressions can be used to greater benefit. Furthermore, while the approximation between individual terms may be less than desired, all parameters $c_{n}$ and $\mu_{n}$ can be adjusted by appropriate curve fitting of creep and relaxation data so that the sums match each other collectively with much greater accuracy than that of each individually paired term. This is in keeping with the practical requirements of actually modeling the behavior of real materials in terms of the entire complex modulus representations without the need to necessarily produce precise values for each of its constituent parameters. The advantage of the Equation (18) is that the approximation on the r. h. s. has a known inverse and an approximate analytical expression can then be obtained by inverting Equations (10) and (16) to yield

(19) $$\psi_{FD}\left(t\right)\;≃\;\underset{\text{initial spring}}{\underset{︸}{\frac{1}{G_{0}}}}\;+\;\underset{\text{final dashpot}}{\underset{︸}{\frac{t^{\beta_{N+1}+1}}{\eta_{N+1}\;\Gamma \left(\beta_{N+1}+1\right)}}}\;+\;\sum_{n=1}^{N}\underset{\text{Kelvin models}}{\underset{︸}{\left[\frac{1}{\eta_{n}\;\Gamma \left(\mu_{n}\right)}\int_{0}^{t}p^{\mu_{n}-1}\;\exp\left(-\;\frac{p}{\tau_{n}}\right)\;dp\right]}}$$

where the first two expressions are exact, but the last is approximate for $\psi_{FD}\left(t\right)$ even though it is the exact inverse of the r. h. s. of Equation (18). The exact expression for the Kelvin model integrals is given in Equation (44).

The isothermal integral fractional derivative constitutive relations are

(20) $$2\;E_{kl}\left(t\right)\;=\;\int_{0}^{t}\psi_{FD}\left(t-t^{\prime }\right)\;\frac{\partial S_{kl}\left(t^{\prime }\right)}{\partial t^{\prime }}\;dt^{\prime }\;=\;\int_{0}^{t}J_{FD}\left(t-t^{\prime }\right)\;S_{kl}\left(t^{\prime }\right)\;dt^{\prime }$$

and they differ from the integer derivative ones only in the definition of the creep and relaxation functions, *i.e.*, by replacing $\psi_{FD}$ with *ψ* in Equation (20).

Expressions similar to Equations (5) through Equation (20) can also be formulated for volumetric constitutive relations for *σ* and *ϵ* resulting in

(21) $$\epsilon \left(t\right)\;=\;\int_{0}^{t}\psi_{FD}^{V}\left(t-t^{\prime }\right)\;\frac{\partial \sigma (t^{\prime })}{\partial t^{\prime }}\;dt^{\prime }\;+\;\int_{0}^{t}\psi_{FD}^{VT}\left(t-t^{\prime }\right)\;\frac{\partial \left[AT(t^{\prime })\right]}{\partial t^{\prime }}\;dt^{\prime }$$

where A is the coefficient of thermal expansion. Using Equations (3) and (4) and substituting Equation (21) into Equation (20) produces a single set of isotropic constitutive relations between $\epsilon_{kl}$ and $\sigma_{kl}$ such that

(22) $$\begin{array}{c} \begin{array}{cc} 2\epsilon_{kl}\left(t\right)\;= & \;\int_{0}^{t}\psi_{FD}\left(t-t^{\prime }\right)\;\frac{\partial [\sigma_{kl}\left(t^{\prime }\right)-\delta_{kl}\;\sigma \left(t^{\prime }\right)]}{\partial t^{\prime }}\;dt^{\prime }\\ & +\;2\delta_{kl}\left\{\int_{0}^{t}\psi_{FD}^{V}\left(t-t^{\prime }\right)\;\frac{\partial \sigma (t^{\prime })}{\partial t^{\prime }}\;dt^{\prime }\;+\;\int_{0}^{t}\psi_{FD}^{VT}\left(t-t^{\prime }\right)\;\frac{\partial \left[AT(t^{\prime })\right]}{\partial t^{\prime }}\;dt^{\prime }\right\} \end{array} \end{array}$$

As can be seen from these isotropic stress-strain relations, the normal strains are related only to normal stresses and shear strains are expressed in terms of only a single corresponding shear stress component, such that

(23) $$\begin{array}{cccc} \epsilon_{\underline{ii}}\; & =\;f_{\underline{ii}}\left[\sigma_{11},\sigma_{22},\sigma_{33},t\right]\; & \text{or}\;\sigma_{\underline{ii}}\; & =\;F_{\underline{ii}}\left[\epsilon_{11},\epsilon_{22},\epsilon_{33},t\right] \end{array}$$

(24) $$\begin{array}{cccc} \epsilon_{ij}\; & =\;f_{\underline{ij}}\left[\sigma_{\underline{ij}},t\right]\; & \text{or}\;\sigma_{ij}\; & =\;F_{\underline{ij}}\left[\epsilon_{\underline{ij}},t\right]\;i\neq j \end{array}$$

These isotropic constitutive relations—and in the next section their anisotropic cousins—are cast in the form of relaxation and/or creep functions, rather than combinations of the latter and viscoelastic Poisson’s ratios (PR). This is due to the fact viscoelastic PRs have been shown to be intrinsic functions of time [42,43,44], stresses and loading conditions and not “pure” material properties such as relaxation functions or moduli [45,46,47,48].

From a practical analysis point of view, the integral constitutive Equations (20) to (22) are always preferable to those expressed as differential operators such as Equation (14) even if the derivatives are of integer order. While the numerical evaluations of convolution integrals are CPU and memory intensive, they are by far more accurate than numerical solutions of high order PDEs. Furthermore, the discretized recurrence relations for such convolution integrals based on only two previous time steps developed in [49] are computationally economical and accurate.

For distinct temperature conditions viscoelastic materials admit the presence of temperature shift functions $a_{T}\left(T\right)$, which for thermorheologically simple materials (TSM) are material property functions of temperature, moisture, degree of cure and aging effects [34,50,51]. An associated reduced time *ξ* can then be defined as

(25) $$\xi \left(x,t\right)\;=\;\int_{0}^{t}a_{T}\left(x,t^{\prime }\right)\;dt^{\prime }$$

which transforms Equation (20) to a single relation in *ξ* instead of *t* for all service temperatures called the master relaxation or creep curve. The volumetric constitutive Equation (21) will have two additional reduced times, one for stresses or strains and the other for thermal expansions. Thus isotropic viscoelastic materials may have three distinct reduced times, although for convenience they are often approximated as all equal to each other, which makes characterization in terms of deviatoric and volumetric master curves possible. More complex nonisothermal behavior has been described in [24,52,53]. During the curing processes the material properties are also dependent on the degree of cure as well as on temperature and moisture content, thus further complicating the modeling of viscoelastic responses [54], but not conceptually altering any of the above results except that the shift function, as well as other parameters, become $a_{T}\left(T,M,\alpha \right)$, ξ(T,M,α), $G_{n}\left(T,M,\alpha \right)$ and $\eta_{n}\left(T,M,\alpha \right)$ where *α* is the degree of cure and *M* the moisture content, and with similar expressions for the volumetric components.

Nonlinear viscoelastic behavior necessitates that creep and relaxation functions are strain dependent, *i.e.*, $\psi =\psi [x,\xi \left(x,t\right),I_{1}\left(x,\xi \right),I_{2}\left(x,\xi \right),I_{3}\left(x,\xi \right)]$, where $I_{m}$ are the first three invariants of the strain tensor $\epsilon_{kl}$ [55]. The nonlinearities introduce additional complications into the analysis and make analytical solutions difficult if not impossible, necessitating numerical finite element formulations. While FT cannot be successfully applied to nonlinear constitutive relations, they can be used in the generalized linear formulation and then nonlinearities can be introduced *a posteriori* through proper interpretation when the entire linear modeling has been formulated and completed.

### 2.3. Anisotropic Relations

For anisotropic viscoelastic materials it is no longer possible to readily separate the stresses and strains into deviatoric and volumetric components, because volumetric stresses are now material property dependent [35]. Material parameters associated with mechanical models, such as $E_{ijkln}$ and $\eta_{ijkln}$ instead of the isotropic ones $E_{n}$ and $\eta_{n}$, must be identified with specific directions. Anisotropic states can then be achieved in three separate ways:

(1) different parametric values $E_{ijkln}$ and $\eta_{ijkln}$ with equal numbers *N* in all directions
(2) same parameters $E_{ijkln}$ and $\eta_{ijkln}$ in all directions but with distinct $N_{ijkl}$, thus generating different numbers of GKM parameters in each direction
(3) combinations of (1) and (2) above

Inherent in such anisotropic behavior is the need to prescribe distinct integral and differential operator equations with property functions which are directionally dependent and which are associated with $\phi_{kl}$, $\psi_{kl}$, $P_{kl}$, $Q_{kl}$, *etc*. In matrix form this can be written as

(26) $$\left\{\epsilon \right\}\;=\;\left[C\right]\;\left\{\sigma \right\}\;+\;\left[C_{T}\right]\;\left\{AT\right\}$$

where the compliance matrices [C] and $\left[C_{T}\right]$ are either a set of integral or differential operators, resulting in FT expressions

(27) $$\left\{\bar{\bar{\epsilon }}\right\}\;=\;\left[\bar{\bar{C}}\right]\;\left\{\bar{\bar{\sigma }}\right\}\;+\;\left[\bar{\bar{C_{T}}}\right]\;\left\{\bar{\bar{AT}}\right\}$$

Consequently, the anisotropic integral constitutive relations incorporating the above features and following the pattern of the isotropic relations of Equations (20) and (21) now read

(28) $$\epsilon_{ij}\left(x,t\right)\;=\;\int_{-\infty }^{t}\psi_{ijkl}\left(x,t,t^{\prime }\right)\;\frac{\partial \sigma_{kl}\left(x,t^{\prime }\right)}{\partial t^{\prime }}\;dt^{\prime }\;+\;\int_{-\infty }^{t}\psi_{ijklT}\left(x,t,t^{\prime }\right)\;\frac{\partial \left[A_{kl}\;T\right]\left(x,t^{\prime }\right)}{\partial t^{\prime }}\;dt^{\prime }$$

with similar expressions for the fractional derivative ones obtained by changing $\psi_{ijkl}$ and $\psi_{ijklT}$ to $\psi_{ijkl}^{FD}$ and $\psi_{ijklT}^{FD}$ respectively. A modification of Equation (19) incorporating anisotropic representations then leads to

(29) $$\begin{array}{c} \begin{array}{cc} \psi_{ijkl}^{FD}\left(t\right)\;\tilde{=}\; & \frac{1}{G_{0}}\;+\;\frac{t^{\beta_{N_{\underline{ijkl}}+1}+1}}{\eta_{N_{\underline{ijkl}}+1}\;\Gamma \left(\beta_{N_{\underline{ijkl}}+1}+1\right)}\\ & +\;\sum_{n=1}^{N_{\underline{ijkl}}}\left[\frac{1}{\eta_{\underline{ijkl}n}\;\Gamma \left(\mu_{\underline{ijkl}n}\right)}\int_{0}^{t}p^{\mu_{\underline{ijkl}n}-1}\;\exp\left(-\;\frac{p}{\tau_{\underline{ijkl}n}}\right)\;dp\right] \end{array} \end{array}$$

where the underscore indicates no summations on the marked indices.

Similarly, associated differential operators can be found by properly interpreting Equation (14) as

(30) $$\begin{array}{c} \begin{array}{cc} {\hat{P}}_{ij}\left(x,t\right)\; & =\;\sum_{n=0}^{r_{\underline{ij}}}{\hat{a}}_{\underline{ij}n}\left(x,t\right)\;D^{\alpha_{\underline{ij}n}}\\ {\hat{Q}}_{ijkl}\left(x,t\right)\; & =\;\sum_{n=0}^{s_{\underline{ijkl}}}{\hat{b}}_{\underline{ijkl}n}\left(x,t\right)\;D^{\beta_{\underline{ijkl}n}}\\ {\hat{Q}}_{ijklT}\left(x,t\right)\; & =\;\sum_{n=0}^{s_{\underline{ijkl}}^{\prime }}{\hat{b}}_{\underline{ijkl}Tn}\left(x,t\right)\;D^{\gamma_{\underline{ijkl}n}} \end{array} \end{array}$$

where the symbol ^ is generic and refers to either integer or fractional derivative representations and for integer derivative characterization $\alpha_{ijkln}=\beta_{ijkln}=\delta_{ijkln}=n$. The anisotropic DE constitutive relations then become

(31) $${\hat{P}}_{\underline{ij}}\left\{\sigma_{\underline{ij}}\right\}\;=\;{\hat{Q}}_{ijkl}\left\{\epsilon_{kl}\right\}\;-\;{\hat{Q}}_{ijklT}\left\{A_{kl}T\right\}$$

Temperature shift functions and reduced times must similarly be directionally identified as

(32) $$\begin{array}{cc} \xi_{ijkl}\left(x,t\right)\; & =\;\int_{0}^{t}a_{Tijkl}\left[x,s,T\left(x,s\right),M\left(x,s\right),\alpha \left(x,s\right)\right]\;ds \end{array}$$

(33) $$\begin{array}{cc} \xi_{ijklT}\left(x,t\right)\; & =\;\int_{0}^{t}a_{TijklT}\left[x,s,T\left(x,s\right),M\left(x,s\right),\alpha_{cure}\left(x,s\right)\right]\;ds \end{array}$$

While in isotropic viscoelasticity there are only two *ψ*s, one $\psi_{T}$, two *ξ*s and one $\xi_{T}$, anisotropic viscoelasticity requires due to symmetry a maximum of 21 $\psi_{ijkl}$ and $\xi_{ijkl}$ each and six $\psi_{ijklT}$ and $\xi_{ijklT}$. The possible presence of even two or three reduced times in isotropic viscoelasticity, much less 27 for anisotropic responses, makes any solution by analytical means totally unmanageable. For effective analytical anisotropic viscoelastic formulations, it is, therefore, necessary to introduce only a single shift function and a single reduced time. This is analogous to what is done in isotropic viscoelasticity where the shift functions for changes in volume and shape and the one for thermal expansions are all equated to each other. Then

(34) $$a_{T}\;=\;a_{Tijkl}\;=\;a_{TijklT}$$

and

(35) $$\xi \left(x,t\right)\;=\;\xi_{ijkl}\;=\;\xi_{ijklT}$$

This simplifies the constitutive relations to

(36) $$\begin{array}{c} \begin{array}{cc} \epsilon_{ij}\left(x,\xi \right)\;= & \;\int_{-\infty }^{\xi }{\hat{\psi }}_{ijkl}\left(x,T_{0},M_{0},\xi -\xi^{\prime }\right)\;\frac{\partial \sigma_{kl}\left(x,\xi^{\prime }\right)}{\partial \xi^{\prime }}\;d\xi^{\prime }\\ & +\;\int_{-\infty }^{\xi }{\hat{\psi }}_{ijklT}\left(x,T_{0},M_{0},\xi -\xi^{\prime }\right)\;\frac{\partial \left[A_{kl}T\right]\left(x,\xi^{\prime }\right)}{\partial \xi^{\prime }}\;d\xi^{\prime } \end{array} \end{array}$$

where $T_{0}$ and $M_{0}$ are the reference temperature and the moisture content respectively [35,49,54,55,56]. It should be noted that the shift function $a_{T}$ and $\hat{\psi }$ itself and consequently the reduced time *ξ* may change their functional definitions drastically from one temperature and moisture region to another as the material undergoes phase changes, as is for instance the case during cure processes where the material property functions become additionally dependent on the degree of cure *α* [54, *i.e.*, $\hat{\psi }\left(x,T,M,\alpha ,\xi -\xi^{\prime }\right)$ or $\hat{\psi }\left(x,T,M,\alpha ,\xi ,\xi^{\prime }\right)$.

Nonlinear responses may again be introduced into the characterization in the same manner as in the isotropic case, *i.e.*,

(37) $$\psi_{ijkl}\;=\;\psi_{ijkl}\left[x,t,T\left(x,t\right),M\left(x,t\right),\alpha \left(x,t\right),I_{1}\left(x,t\right),I_{2}\left(x,t\right),I_{3}\left(x,t\right)\right]$$

Similar functional expressions can be generated for $\psi_{ijklT}$, $\phi_{ijkl}$ and $\phi_{ijklT}$. Such nonlinear representations, of course, turn the material property parameters $\mu_{ijkln}$, $\eta_{ijkln}$ and $\tau_{ijkln}$ of Equations (19) and (29) into functions of the variables contained in the preceding square bracket defining *ψ*. Specific isotropic examples have been formulated in [57] and extended to anisotropic viscoelasticity in [55].

Practical computational questions need to be raised about the FD characterization involving creep or relaxation functions. Neither the approximate Equations (19) and (29) nor the corresponding exact ones can be directly analytically integrated except by (asymptotic) series expansions for each $\mu_{ijkln}$, thereby increasing computational times for FEM solutions. It must be remembered that this solution process is inherently costly since these integrals or series must be repeatedly evaluated at every node point and at each time step in every direction.

One must, therefore, turn to an alternative but equally valid characterization for both isotropic and anisotropic viscoelastic materials in terms of compliances rather than creep functions. For anisotropic materials, Equation (16) can be rewritten as

(38) $$ı\;\omega \bar{\bar{\phi_{ijkl}^{FD}}}\;=\;\frac{1}{ı\;\omega \bar{\bar{\psi_{ijkl}^{FD}}}}\;=\;\bar{\bar{G_{ijkl}^{FD}}}\;=\;\frac{1}{\bar{\bar{J_{ijkl}^{FD}}}}$$

The constitutive Equation (28) in terms of compliances are

(39) $${\hat{\epsilon }}_{ij}\left(x,\xi \right)\;=\;\int_{-\infty }^{\xi }{\hat{J}}_{ijkl}\left(x,\xi -\xi^{\prime }\right)\;\frac{\partial \sigma_{kl}\left(x,\xi^{\prime }\right)}{\partial \xi^{\prime }}\;d\xi^{\prime }\;+\;\int_{-\infty }^{\xi }{\hat{J}}_{ijklT}\left(x,\xi -\xi^{\prime }\right)\;\frac{\partial \left[A_{kl}T\left(x,\xi^{\prime }\right)\right]}{\partial \xi^{\prime }}\;d\xi^{\prime }$$

with similar relations in the *ξ* space replacing Equations (14) and including both isotropic and anisotropic ID and FD representations. The inverse of the FD compliance $\bar{\bar{J_{ijkl}^{FD}}}$ for time dependent properties in the *ξ* space defined by Equations (10) and (18) is

(40) $$\begin{array}{c} \begin{array}{cc} J_{ijkl}^{FD}\left(x,\xi \right)\;\tilde{=} & \;\frac{\delta (\xi )}{G_{0ijkl}\left(x,\xi \right)}\;+\;\frac{\xi^{\beta_{N_{\underline{ijkl}}+1}}}{\eta_{N_{\underline{ijkl}}+1}\left(x,\xi \right)\;\Gamma \left(\beta_{N_{\underline{ijkl}}+1}\right)}\;\\ & +\;\sum_{n=1}^{N_{\underline{ijkl}}}\left\{\frac{\xi^{\left(-1+\mu_{\underline{ijkl}}n\right)}}{\eta_{\underline{ijkl}n}\left(x,\xi \right)\;\Gamma \left(\mu_{\underline{ijkl}n}\right)}\;\exp\left(-\int_{0}^{\xi }\frac{d\xi^{\prime }}{\tau_{\underline{ijkl}n}^{\prime }\left(x,\xi^{\prime }\right)}\right)\right\} \end{array} \end{array}$$

where $1/\tau_{ijkln}^{\prime }=c_{ijkln}$ and the $\tau_{ijkln}^{\prime }$ do not necessarily represent actual relaxation times. For time independent nonhomogeneous anisotropic properties, the compliances reduce to

(41) $$\begin{array}{c} \begin{array}{cc} J_{ijkl}^{FD}\left(x,\xi \right)\;\tilde{=} & \;\frac{\delta (\xi )}{G_{0ijkl}\left(x\right)}\;+\;\frac{\xi^{\beta_{N_{\underline{ijkl}}+1}}}{\eta_{N_{\underline{ijkl}}+1}\left(x\right)\;\Gamma \left(\beta_{N_{\underline{ijkl}}+1}\right)}\;\\ & +\;\sum_{n=1}^{N_{\underline{ijkl}}}\left\{\frac{\xi^{\left(-1+\mu_{\underline{ijkl}n}\right)}}{\eta_{\underline{ijkl}n}\left(x\right)\;\Gamma \left(\mu_{\underline{ijkl}n}\right)}\;\exp\left(\frac{-\;\xi }{\tau_{\underline{ijkl}n}^{\prime }\left(x\right)}\right)\right\} \end{array} \end{array}$$

The constitutive Equation (39) are considerably more tractable for analytical and computational analyses based on either isotropic or anisotropic materials, since they involve single time integrals, rather than the double ones needed for the *ψ* or *φ* relations. The use of moduli or compliances in the FD relations offers minimal computational penalties as it entails almost identical computational time and labor, except for the extra $\xi^{\mu -1}$ in the series terms, when compared to the integer derivative characterization.

The integer derivative constitutive Equation (18) are recoverable from Equations (19) and (20) by setting $\beta_{N_{ijkl}+1}=\mu_{ijkln}=1$ for $1\leq n\leq N_{ijkl}$.

## 3. Discussion

Three of the important problem areas associated with viscoelastic constitutive relations and stress-deformation analyses are:

(a) Performing “simple” experiments for which analytic solution can be formulated and evaluated
(b) Curve fitting of actual creep and/or relaxation data by least square or other methods in order to determine modulus, creep function or compliance parameters
(c) Inversion of FT or LT expressions for moduli, stresses and deformations

Curve fitting of moduli, *etc*. expressions can be carried out in either the real time space *t* or with FT in the frequency domain *ω* [1,2,3]. Values of the parameters $N_{ijkl}$ are selected *a priori* in order to achieve a preassigned accuracy of fit. For the ID representation there are only two sets of other parameters to be determined for each *n* ($1\leq n\leq N_{ijkl}$), namely $\eta_{ijkln}$ and $\tau_{ijkln}$, while for the FD relations a third set $\mu_{ijkln}$ or $\beta_{ijkln}$ must be added.

It can be readily seen from Equations (10), (19) and (41) that these expressions contain nonlinear functional dependencies on *τ*s, *β*s and *μ*s. (Changing the *η*s to their inverses results in their linear contributions and, consequently, they present no computational problems.) For isotropic ID constitutive relations, Schapery [58] has suggested that an approximation be applied to the $\tau_{n}$ by setting each equal to $10^{2n}$ and then leaving only the $1/\eta_{n}$ to be determined by the fit. The same scheme is extended here to anisotropic ID relations. Experience shows that by proper selection of $N_{ijkl}$ any reasonable accuracy of fit can be attained, but for assumed *τ* values the *N*s may be roughly twice as large as those corresponding to the correct *τ* evaluations, *i.e.*, twice as many Prony series [59] terms. Under the Schapery approximation, the *τ*s loose their physical meaning as relaxation times, but the modulus and/or creep functions maintain their accurate representations and physical meaning *in toto*, while the separate parameters do not. This represents no loss of generality in the physical definitions or actual evaluations by curve fit of experimental data of the entire modulus expressions [60,61,62,63].

Similarly, if one extends this idea to anisotropic FD relations, then approximate values can be generated for the exponents by setting $0<\delta_{n}≃\left|n\pm {\tilde{a}}_{n}\right|$ and $0<\mu_{n}≃{|n\pm \tilde{b}}_{n}|$ and where ${\tilde{a}}_{n}$ and ${\tilde{b}}_{n}>0$ can be adjusted for a “best” fit of the experimental data. This greatly simplifies the procedure for determining $1/\eta_{n}$ if additionally the same Schapery approximation is applied to the relaxation times, *i.e.*, $\tau_{n}=\tau_{n}^{\prime }=O\left(10^{2n}\right)$.

The ID representations lead to integrable analytical expressions for *ψ* and *φ* and even though their values of the *N*s may possibly be up to two to three times as large as the comparable ones for the FDs, the general computational time is considerably shorter. However, as was shown in the previous section, in order to avoid the second time integral needed for the $\psi_{ijkl}$ inversions in the FD forms, one must use the compliance form of the constitutive relations as shown in Equation (38). This still necessitates FT inversions to determine compliances as time functions. Such FT inversions can be accomplished by

1. exact procedures, such as table look up or analytical evaluation of the *ω* FT inversion integrals
2. numerical procedures, such as FFT [41]
3. approximate procedures, such as those devised in [58], given by Equation (45) and limited to quasi-static problems where inertia and body forces are negligible
4. exact for k=∞, but approximate for k<∞ LT inversions due to Post [64] as

(42) $$f\left(x,t\right)\;=\;\lim_{k\to \infty }{\left[\frac{{\left(-1\right)}^{k}}{k!}\;p^{k+1}\;\frac{\partial^{k}\bar{f}\left(x,p\right)}{\partial p^{k}}\right]}_{p=k/t}$$

where *k* are positive integers. Note that when the transform integrals exist, the relationship between LTs and FTs is given by

(43) $$\bar{f}\left(x,p\right)\;=\;\int_{0}^{\infty }f\left(x,t\right)\;\exp\left(-p\;t\right)\;dt\;=\;{\left.\bar{\bar{f}}\left(x,\omega \right)\right|}_{p=ı\;\omega }=\;{\left.\underset{\text{= half interval FT}}{\underset{︸}{\int_{0}^{\infty }\;f\left(x,t\right)\;\exp\left(-ı\;\omega \;t\right)\;dt}}\right|}_{p=ı\;\omega }$$

Table look up and/or formal inversions through integration are, of course, the approaches of choice. Unfortunately, these work only for limited classes of LT and FT functions. In principle FFT always work but, as has been shown in [65] they are computationally intensive even on supercomputers because in order to obtain accurate FT inversion values one must sweep some 13 or more decades of *ω*, thus making the process computationally very costly. Finally, the approximate inversion scheme of Schapery based on producing inversions by multiplying the FT by ıω and then setting ıω=p=.5/t in the transform leads to simple procedures. Recalling that at the reference temperature ξ=t, the exact and approximate inverses of the r. h. s. of Equation (18) are given by

(44) $$\begin{array}{cc} \text{exact}\;F_{1n}\left(\xi \right)\; & =\;\frac{\xi^{\mu_{n}-1}\;\exp\left(-\xi /\tau_{n}^{\prime }\right)}{\Gamma (\mu_{n})} \end{array}$$

(45) $$\begin{array}{cc} \text{approximate}\;F_{2n}\left(\xi \right)\; & ≃\;\frac{p}{{\left(p+1/\tau_{n}^{\prime }\right)}^{\mu_{n}}}{|}_{p=.5/\xi } \end{array}$$

**Figure 4:**
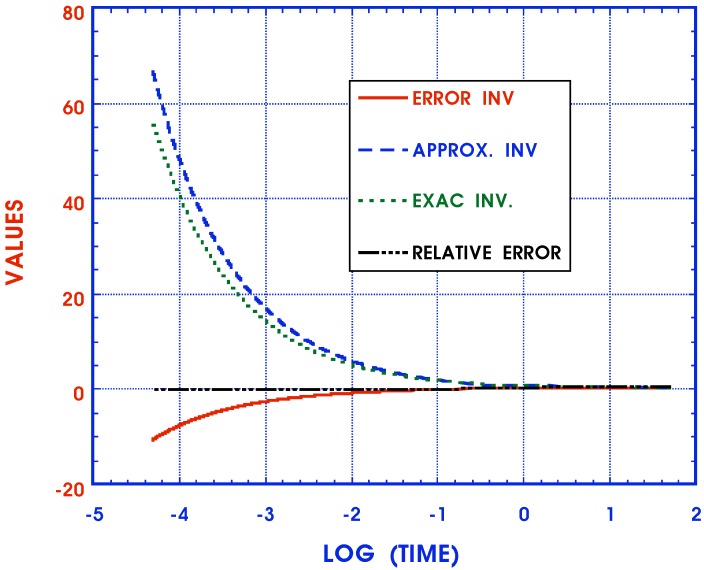
Fractional derivative inverse.

These functions together with the absolute error $F_{1n}\left(\xi \right)-F_{2n}\left(\xi \right)$ are displayed in Figure 4 . (The relative error cannot be shown because the exact solution tends to zero for larger values of *ξ*.) It is seen that the approximate inverse, while simple, is not very accurate. Equations (44) and (45) deal with only a single term of the Equation (40) representing the FD anisotropic compliances $J_{ijkl}^{FD}\left(\xi \right)$. The cumulative effect of such errors makes the Schapery approximate inversion scheme unacceptable in this instance.

Accuracy limitations of Schapery’s approximate inversion method when applied to dynamic isotropic viscoelastic problems has been previously pointed out in [66,67,68], among others. Although the method has found favor with much justification in quasi-static viscoelastic problems, this appears to be the first time that Schapery’s approximate inversion of a non-Prony series complex compliance itself is seen to also yield undesirable time functions.

In [69] the simple quasi-elastic and more involved self-consistent approximations for evaluating convolution integrals are compared. The reference’s conclusion is that the self-consistent method is more accurate, but yields more complicated results. The quasi-elastic method simply stated is

(46) $$\sigma_{ij}\left(\xi \right)\;=\;\int_{0}^{\xi }E_{ijkl}\left(\xi -\xi^{\prime }\right)\;\epsilon_{kl}\left(\xi^{\prime }\right)\;d\xi^{\prime }\;≃\;E_{ijkl}\left(\xi \right)\;\epsilon_{kl}\left(\xi \right)$$

still requires knowledge of the inverses of ${\bar{\bar{E}}}_{ijkl}$ and ${\bar{\bar{\epsilon }}}_{kl}$, but avoids the need for costly convolution integral evaluations. However, it is applicable only intermittently over relatively short time intervals and unfortunately, it is adequate only for simple classes of functions, such as single power laws, and cannot begin to cope with the complicated set of functions describing real material relaxation moduli [70]. The self-consistent approach leads to complicated results, even when tested against relatively simple functions, and is of limited practical use for real materials with the usual series expansions for their viscoelastic moduli.

More accurate, but also considerably more cumbersome, approximate inversion schemes are available [71]. Other pertinent works on approximate integral transform inversions may be found in [72,73,74,75].

Another possibility is the expansion of the complex moduli or compliances for both ID and FD characterizations in terms of a Lorenz series [3], such that

(47) $${\bar{\bar{J}}}_{ijkl}\left(\omega \right)\;=\;\sum_{n=0}^{\infty }\left[\frac{A_{ijkln}}{{\left(ı\;\omega \right)}^{n}}\;+\;B_{ijkln}\;{\left(ı\;\omega \right)}^{n}\right]$$

where the $A_{ijkln}$ and $B_{ijkln}$ coefficients are determined by curve fitting experimental data. Even when truncated, the first sum gives excellent results for short times, ω≫1, and the second one does the same for long times when ω≪1. However, both sums must be used in the entire space 0<t<∞, except as noted in the relatively narrow bands at either time end, making this approach very cumbersome when combined with FEM for isotropic or anisotropic viscoelasticity.

The selection between FD and ID characterization then comes down to a choice between (a) fitting and using more complicated FD stress-strain laws with fewer individual terms but three parameters per term, and (b) simpler ID ones with two parameters per term but needing more terms in their series. In the final analysis, the ease with which the ID Prony series parameters can be generated from real material data in either the *t* or *ω* domains outweighs any advantages the FD representations may offer with its relatively shorter sums. A case in point is the work by [76] comparing the performance of a single ID Kelvin body with a 3- and 4-parameter FD model, showing marked improvements in accuracy of fit for FD models over ID ones for rubber and acrylic materials. However, as seen in Figure 6 , more complex real materials such as composites require ID characterizations with values for each $N_{ijkl}$ of approximately 30, *i.e.*, about 61 Prony series parameters. For the corresponding ID and FD models in this example, the $N_{ijkl}$ are 29 and 10 with maximum errors of fit of 0.056 % and 10.15 % respectively. The $N_{ijkl}$ for FD representations can, of course, be increased but this defeats the primary purpose of their use. In the final analysis, the choice between ID and FD characterization has to be examined on a case by case basis for specific problem solutions, particularly those involving highly computationally intensive finite element approaches.

**Figure 5:**
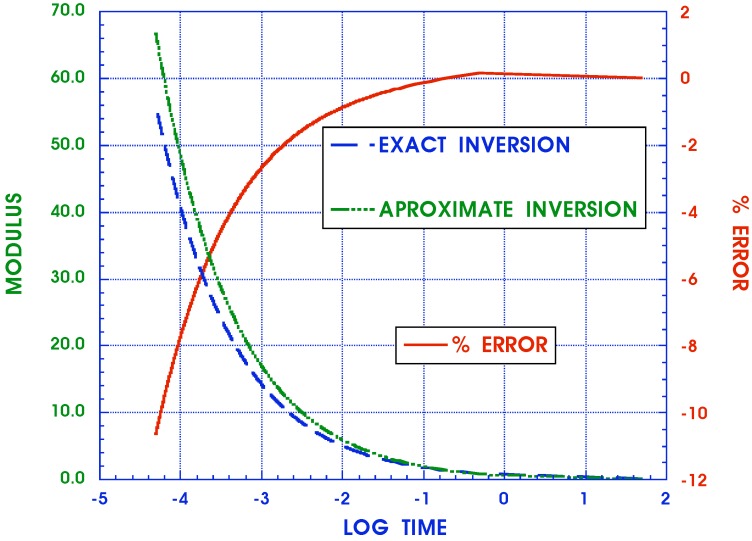
Comparison of ID and FT modeling of $1/{\left(p+c\right)}^{\mu }$.

**Figure 6:**
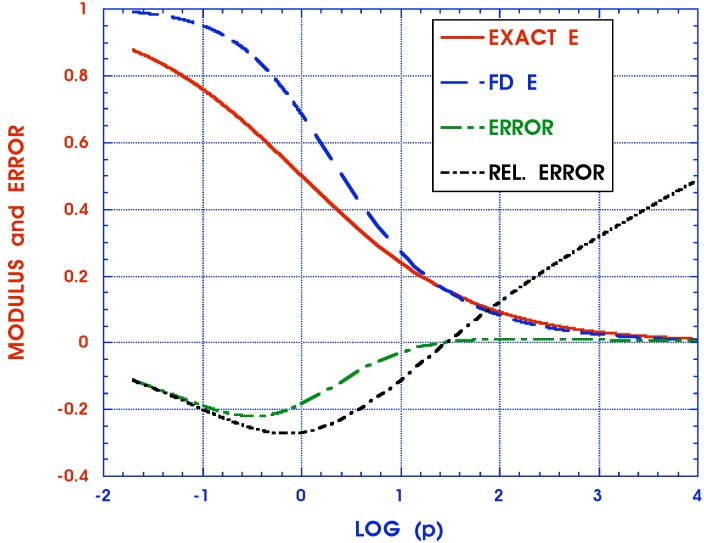
Laplace transform modulus.

The requirement for relatively large $N_{ijkl}$ values for both ID and FD representations make the FD characterizations less attractive and modeling with the relatively simpler ID integral constitutive relations is less computationally intensive. Furthermore, for large scale engineering problems requiring upward of 10,000 FEA nodes, integral transforms are prohibitive and real time or reduced time *ξ* integrations need to be undertaken.

It should be further noted that while the area of highest percentage relative error is in the long time domain (small p values), the continuing mismatch between the actual and FD relaxation moduli spans over three initial time decades which are of primary interest. Because of the hereditary nature of the constitutive relation time integrals, these early time errors propagate throughout the entire time regime and adversely influence stress and displacement evaluations at all times.

Figure 6 represents comparisons for only one direction with normalized relaxation moduli and is typical of what is seen in other directions for anisotropic viscoelastic materials, except for adjustments in the fully relaxed modulus values and in the time scales.

**Figure 7:**
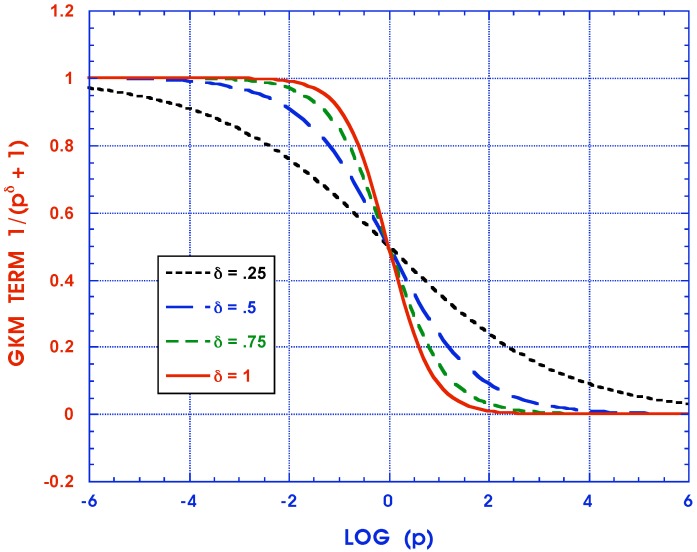
The influence of *δ*.

**Figure 8:**
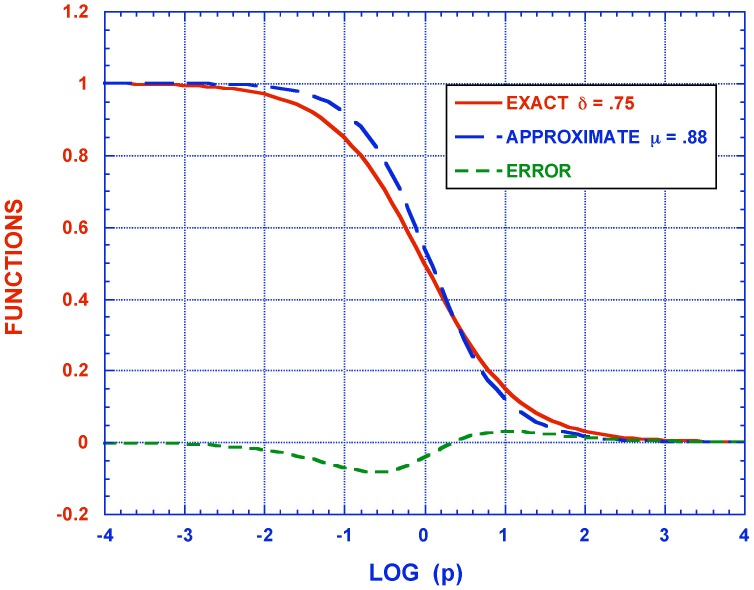
Exact and approximate FD.

In Figure 7 the approximate Kelvin model function of Equation (18) is displayed for a number of δ≤1 values. As can be readily seen these terms are sensitive to the exponent over the entire range of Laplace transform variable *p* and therefore correspondingly over the entire time range -∞≤t≤∞. Figure 8 exhibits the exact curve ${\left(p^{\delta }+1\right)}^{-1}$ and its approximate representation by ${\left(p+1\right)}^{-\mu }$ for specific, but representative, values of δ=0.75 and μ=0.88. The approximate representation mimics the exact one quite well with an error of of less than 0.1 over the entire time range. It must, of course, be remembered that experimental data for viscoelastic material properties is customarily in a comparable scatter range.

**Figure 9:**
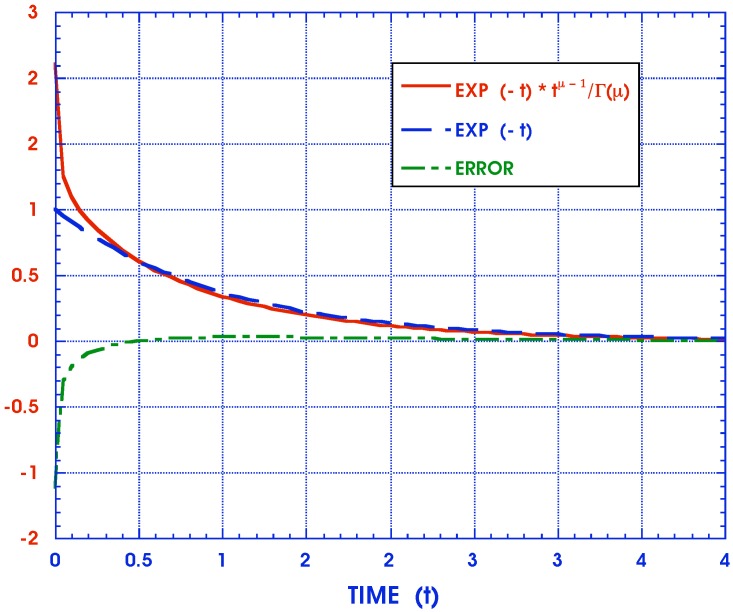
Comparison of ID and approximate FD.

Figure 9 depicts the time functions associated with an ID and an approximate FD (δ=0.88) single Kelvin mode for τ=1. It is readily seen that agreement between the two models is excellent, *i.e.*, error ≅0, except in the neighborhood of the origin t=0. This is due to the fact that for a single Kelvin model

(48) $$\lim_{t\to 0}\left\{\exp\left(-\frac{t}{\tau }\right)\right\}\to 1\;\text{and}\;\lim_{t\to 0}\left\{\frac{\exp\left(-t/c\right)\;t^{\mu -1}}{\Gamma (\mu )}\right\}\to \infty \;0<\mu <1$$

For materials characterized by a sum of approximate FD models,

(49) $$\lim_{t\to 0}\left\{\sum_{n=1}^{N}\;J_{n}\;\frac{\exp\left(-t/c_{n}\right)\;t^{\mu_{n}-1}}{\Gamma (\mu_{n})}\right\}\;=\;\lim_{t\to 0}\left\{\sum_{n=1}^{N}J_{n}\;F_{n}\left(t\right)\right\}\;\to \;\infty \;0<\mu_{n}<1$$

the singularity still cannot be removed even through proper choices of the $J_{n}$, $c_{n}$ and $\mu_{n}$ are made to minimize the error of fit for $\sum_{n=1}^{N}J_{n}F_{n}\left(t\right)$ elsewhere at 0<t≤∞, since no value of $J_{n}\neq 0$ can correct or ameliorate the discrepancy at the time origin when for any one *n* $\mu_{n}<1$.

On the other hand, if the Riemann–Liouville condition of $ℜ(\delta_{n})>0$ is enforced and $\mu_{n}>1$, then

(50) $$\lim_{t\to 0}\left\{J_{n}\;F_{n}\left(t\right)\right\}\;\to \;0\;1<n<N$$

For the Prony series representations in both real time *t*-space and reduced time *ξ* space, the constitutive relations can be transformed into ordinary instead convolution integrals, *i.e.*,

(51) $$\begin{array}{c} \begin{array}{cc} \int_{-\infty }^{\xi (x,t)}\;\;\;\exp\left(-\frac{\xi \left(x,t\right)-\xi^{\prime }\left(x,t^{\prime }\right)}{\tau_{0n}}\right) & \hat{f}\left[\xi^{\prime }\left(x,t^{\prime }\right)\right]\;d\xi^{\prime }\;\to \;\\ & \exp\left(-\frac{\xi (x,t)}{\tau_{0n}}\right)\;\;\int_{-\infty }^{\xi (x,t)}\;\;\;\exp\left(\frac{\xi^{\prime }\left(x,t^{\prime }\right)}{\tau_{0n}}\right)\hat{f}\left[\xi^{\prime }\left(x,t^{\prime }\right)\right]\;d\xi^{\prime } \end{array} \end{array}$$

so as to avoid CPU intensive computations [77]. Unfortunately, no such transformations have been derived for FD constitutive relations.

It is worthy to note that from a practical point of view either FD or ID are use to characterize the same material behavior and are obtained from the same experimental stress, strain and time data by fitting either or both appropriate analytical expressions to the latter. The accuracy of these fits depends on the number of terms used in each truncated series and on the scatter of the experimental data.

The least square fits used to determine parameters for the truncated FD and ID series lead to nonlinear transcendental relations in the first instance and to linear algebraic ones for the Prony series [60,61,62,63]. Comparisons of the goodness of fit for the two approaches can only be made only in a case by case approach for a specific material and a given set of experimental data.

## 4. Conclusions

While sufficiently accurate approximate procedures have been formulated to reasonably characterize real anisotropic materials with generalized integral and differential viscoelastic fractional derivative constitutive relations, their use with or without approximate characterization protocols requires functional complexity. Although integer derivative representations require possibly twice or three times as many terms in the expressions defining viscoelastic moduli, the relative simplicity of the resulting Prony series leads to computationally efficient anisotropic viscoelastic constitutive relations in real time as well as in FT frequency domains. In both fractional and integer derivative use, characterizations by integral rather than differential constitutive laws remain operationally preferred models because of their higher computational accuracies.
